# Biomechanical analysis of gait initiation in stroke survivors based on a modified phase segmentation method

**DOI:** 10.3389/fneur.2026.1716860

**Published:** 2026-02-09

**Authors:** Liling Liu, Wei Wang, Hui Wei, Shouwei Yue

**Affiliations:** 1 Rehabilitation Center, Qilu Hospital of Shandong University, Jinan, Shandong, China; 2 Department of Rehabilitation Medicine, The First Affiliated Hospital of USTC, Division of Life Science and Medicine, University of Science and Technology of China, Hefei, Anhui, China

**Keywords:** anticipatory postural adjustment, center of pressure, gait initiation, rehabilitation, stroke

## Abstract

**Purpose:**

This study aims to explore the characteristics of gait initiation in stroke patients with hemiplegia across various phases by proposing a refined gait initiation phase segmentation method tailored to this population. Additionally, the study investigates the correlation between gait initiation parameters and clinical assessment scales to evaluate their clinical value in development of functional assessment and rehabilitation strategies.

**Methods:**

A total of 34 patients with hemiparetic stroke and 34 age- and sex-matched healthy controls participated in the study. All participants performed gait initiation with each limb three times. Ground reaction force, center of pressure (COP), and kinematic data were recorded using a gait analysis system. The gait initiation process was divided into four phases based on COP trajectory and movement features. Step length, step width, initial paretic limb loading, duration and COP displacement across various phases, and the peak (*F*_xmax_) and impulse (Impulse_x_) of the anteroposterior ground reaction force for each limb were calculated. Stroke patients also underwent clinical assessments.

**Results:**

Stroke patients exhibited increased step width and total initiation duration (*p* < 0.05); reduced step length, single-support time of paretic limb, COP displacement in anteroposterior (DCOPX) and mediolateral (DCOPZ) directions during both the first phase (P1) and single-support phase (P3), paretic limb loading, *F*_xmax_ and Impulse_x_ (*p* < 0.05). Compared to non-paretic limb initiation, paretic limb initiation showed shorter duration and reduced DCOPZ of the second phase (P2), as well as longer single-support time and total initiation duration (*p* < 0.05). In addition, the paretic limb generated lower *F*_xmax_ and Impulse_x_ than the non-paretic limb (*p* < 0.05). DCOPX, *F*_xmax_, and Impulse_x_ were significantly correlated with clinical assessments to varying degrees (*p* < 0.05). Overall, greater COP displacement, *F*_xmax_, and Impulse_x_ were associated with better clinical function, particularly in the paretic limb.

**Conclusion:**

Refined phase segmentation provided additional insight: impaired anticipatory postural adjustment capacity during gait initiation after stroke was reflected by prolonged P2 and reduced COP displacement in P1. Biomechanical parameters of gait initiation may serve as objective indicators of motor function and inform rehabilitation strategies in stroke patients.

## 1 Introduction

Gait impairment is a common consequence of stroke, and restoring walking ability remains a primary goal in post-stroke rehabilitation (1, 2). Previous research has focused on steady-state walking, while gait initiation remains relatively underexplored (2–4). Gait initiation represents a transitional phase from static standing to cyclic walking. It is characterized by postural adjustments, inherent instability, and greater attentional demands than steady-state walking, thereby posing an increased risk of falls (5–7). This risk is particularly evident among individuals with neurological impairments, such as stroke survivors, who often demonstrate impaired postural control and are more likely to fall during transfers (8, 9). Therefore, further investigation into gait initiation in this population is essential for understanding its underlying mechanisms and informing targeted rehabilitation and fall prevention strategies.

The gait initiation process begins with an anticipatory postural adjustment (APA) phase, from the onset to the toe-off of the leading limb, and extends to the toe-off of the trailing limb (4, 10, 11). The APA phase comprises two biomechanically distinct stages. Initially, body weight shifts toward the leading limb as the tibialis anterior muscles activate and the soleus muscles relax (12). The COP shifts posterior-laterally toward the leading limb, which generates a ground reaction force propelling the body forward and toward the trailing limb (13). Subsequently, weight is transferred to the trailing limb, allowing the leading limb to clear the ground (4, 10).

Current research in stroke patients has primarily focused on the APA phase, with relatively few studies examining the entire gait initiation process (4, 14–16). Stroke patients frequently demonstrate delayed gait initiation and reduced posterior and lateral COP displacement toward the leading limb during the APA phase (15–17), which may result in decreased forward momentum of the center of mass (COM) (13), ultimately leading to shorter step length and slower gait initiation velocity (15, 17, 18). Bensoussan et al. (16) and Tokunoa and Eng (18) confirmed the reduction in propulsion in the paretic limb and its correlation with gait initiation velocity in stroke patients. Moreover, stroke survivors often exhibited asymmetric gait initiation, with COP and COM trajectories more closely resembling those of healthy controls when initiating with the paretic limb (15).

Additionally, limited studies examining the full gait initiation process segmented the gait initiation process based on movement characteristics (such as toe off of each limb, striking of the leading limb), dividing it into the APA phase, single support phase, and double support phase (15, 16). This segmentation method treated the APA phase as a single unit, although the APA phase comprises two distinct weight shift processes. Such aggregation may obscure critical biomechanical characteristics specific to each sub-phase, thereby limiting analytical precision.

In this context, further investigation into the entire gait initiation process in stroke survivors, along with establishing refined phase division methods, appears both meaningful and necessary. Thus, the primary objective of this study is to refine the phase division of gait initiation and to examine gait initiation characteristics across distinct phases throughout the entire initiation process in stroke patients. This study also examines different gait initiation performances between the paretic and non-paretic limb initiation conditions, considering the lower-limb asymmetry of patients with hemiplegia (15, 16).

In addition, few studies explored the association between gait initiation parameters and clinical assessments, which may help identify clinically meaningful indicators and inform targeted rehabilitation strategies. To the best of our knowledge, one study has reported significant correlations between maximal posterior COP displacement during the APA phase and paretic-limb latency to peak COP displacement with clinical outcomes (19). However, the associations between other key gait initiation parameters and widely used clinical assessment tools remain unclear. Therefore, the second aim of this study was to examine the associations between gait initiation parameters and clinical assessments reflecting distinct functional domains, including lower limb motor impairment, functional mobility, and walking endurance.

The refined phase division method proposed in this study may help uncover more detailed gait initiation characteristics. Furthermore, gait initiation parameters are expected to correlate with clinical assessments, potentially offering meaningful insights for improving clinical evaluation and informing rehabilitation strategies.

## 2 Methods

### 2.1 Participants

Thirty-four stroke survivors with hemiplegia and 34 age- and gender-matched healthy controls were included in this study.

Inclusion criteria for the stroke group were as follows: participants were required to ① be at least 18 years of age; ② have a first-ever, confirmed diagnosis of stroke; ③ present with unilateral limb paralysis; ④ be ≥14 days post-stroke at enrollment; ⑤ be able to follow simple verbal instructions; ⑥ walk independently without the use of assistive devices or external support; and ⑦ initiate gait independently using both the paretic and non-paretic limbs without physical assistance.

Exclusion criteria for the stroke group included: (1) bilateral cerebral hemispheric lesions or any infratentorial (cerebellar or brainstem) lesion; (2) severe cognitive impairment that significantly hindered test completion; and (3) other conditions potentially affecting gait, such as unilateral neglect, apraxia, visual field deficits, vestibular disorders, musculoskeletal disorders, spinal diseases, other neurological disorders, or the use of medications known to impair balance and gait.

Considering the potential influence of gender and age on gait initiation, the control group was strictly matched to the hemiplegic group by gender and within an age difference of no more than 3 years, to ensure group comparability. The exclusion criteria for the control group were consistent with those applied to the stroke group, as previously described.

This study was approved by the Institutional Ethics Committee (KYLL-202506-014-1), and written informed consent was obtained from all participants. Participant characteristics for both groups are summarized in Table 1.

**Table 1:** General characteristics for individuals in the stroke and control groups.

Characteristics	Stroke group (*n* = 34)	Control group (*n* = 34)	*t* value	*p*-value
Sex (female/male), *n*	7/27	7/27	–	–
Age (years), mean ± SD	48.26 ± 12.23	48.15 ± 12.57	−0.039	0.969
Height (cm), mean ± SD	168.62 ± 7.28	168.56 ± 8.08	−0.032	0.975
Weight (kg), mean ± SD	71.83 ± 11.71	70.63 ± 11.87	−0.421	0.675
Disease type (ischemic/hemorrhagic), *n*	25/9	–	–	–
Hemiparesis side (right/left), *n*	17/17	–	–	–
Time post-stroke (months), median (IQR)	2.33 (1.29, 4.23)	–	–	–
FMA-LE score (max 34), mean ± SD	26.32 ± 4.82	–	–	–
TUG time (seconds), median (IQR)	19.29 (11.21, 32.13)	–	–	–
6MWT (meters), median (IQR)	180.00 (76.73, 388.38)	–	–	–

FMA-LE, Fugl-Meyer Assessment for the Lower Extremity; TUG, Timed Up and Go Test; 6MWT, 6-Minute Walk Test; *n*, Number; SD, Standard deviation; IQR, Interquartile range. Sex distribution was matched between the control and hemiplegia groups.

### 2.2 Experimental set-up

**Figure 1:**
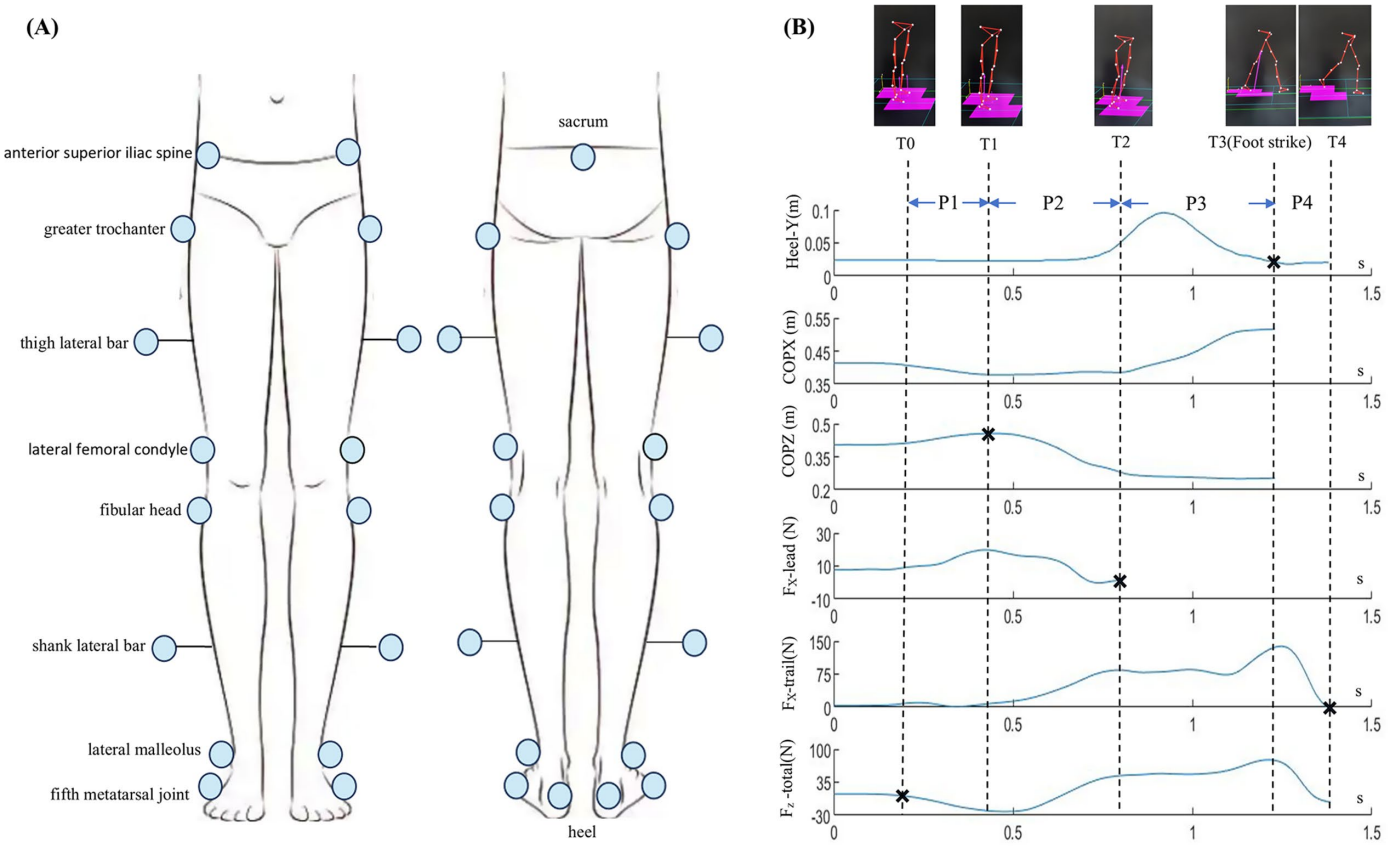
**(A)** Schematic illustration of retroreflective marker placement (anterior and posterior views). **(B)** Four gait initiation phases (P1–P4) defined by five temporal events: P1 (T0–T1), from T0 (onset of initiation) to T1 (COP most lateral toward the swing limb); P2 (T1–T2), from T1 to T2 (leading limb toe-off); P3 (T2–T3), from T2 to T3 (leading limb strike); P4 (T3–T4), from T3 to T4 (trailing limb toe-off). COPX and COPZ denote anteroposterior and mediolateral COP displacement, respectively. *F*_x_-lead and *F*_x_-trail denote anteroposterior forces of the leading and trailing limbs, respectively. Heel-Y denotes the vertical trajectory of the leading heel.

A three-dimensional motion capture and analysis system (BTS Bioengineering S.p.A., Milan, Italy), equipped with six infrared cameras and two force plates, was used to collect kinematic and kinetic data. A total of 19 retro-reflective markers were attached to the lower extremities following the Simple Davis Heel protocol (20) (Figure 1A). In this study, only foot markers were used to calculate step length, step width, and the timing of foot contact of the leading limb. Two force plates (60 × 40 cm) were embedded side by side in a straight walkway approximately 7 m long and 1 m wide. This setup allowed for the separate recording of ground reaction forces for each foot and combined COP trajectories. The motion capture system and force plates were synchronized, sampling at 100 Hz and 200 Hz, respectively.

### 2.3 Test protocol

#### 2.3.1 Gait initiation test

Participants stood barefoot with each foot on a separate force plate in a natural, comfortable posture, with the feet placed approximately shoulder-width apart and aligned in the anteroposterior direction to avoid one foot being positioned ahead of the other. Participants were instructed to gaze straight ahead and were not informed in advance which leg would be used to initiate gait. Gait initiation was triggered by a standardized verbal cue, in which participants were instructed: “When you hear the command ‘initiate with the right (or left) leg’, start walking forward at your comfortable pace when you feel ready.” Based on commonly used protocols in prior studies and considering patient fatigue, each participant performed at least three trials with each leg as the leading limb, with the stepping side randomized. Prior to formal testing, practice trials were conducted for familiarization. All trials were supervised by a researcher to ensure participant safety without physical contact. Rest periods of approximately 2 minutes were provided between trials, and additional breaks were allowed if needed. In addition, leading-limb preference was assessed prior to formal testing; most stroke participants preferred initiating gait with the paretic limb, whereas most healthy controls preferred initiating with the left limb.

#### 2.3.2 Clinical assessment

The stroke group also underwent clinical assessments. Three assessment tools were selected to reflect distinct functional domains: Fugl-Meyer Assessment for the Lower Extremity (FMA-LE) for lower limb motor impairment, the Timed Up and Go (TUG) test for functional mobility, and 6-Minute Walk Test (6 MWT) for walking endurance. All tools have demonstrated good reliability and validity in stroke population (21–23).

### 2.4 Data analysis

#### 2.4.1 Selected parameters

As noted in the introduction, the movement-based phase division method for gait initiation overlooks the fact that the COP shifts in different directions during the early and late stage of the APA phase, which may obscure critical information.

To ensure a more detailed analysis of APA characteristics, this study employed a classification approach that integrates both movement features and COP trajectory characteristics. Specifically, based on the previously established movement-based method (15, 16), the APA phase was further subdivided into two subphases according to the direction of COP displacement. Accordingly, the gait initiation process was divided based on five temporal events, defined as follows:

T0 (onset of movement): defined as the point at which the combined mediolateral force component from both force plates changed by at least 5 N for a minimum duration of 50 ms.

T1 (COP to most lateral): the moment when the COP reached its most lateral position toward the leading limb.

T2 (leading limb toe-off): the moment when the ground reaction force under the leading limb approached zero.

T3 (leading limb strike): the instant of initial ground contact of the leading limb, determined from the foot trajectory.

T4 (trailing limb toe-off): the moment when the ground reaction force under the trailing limb approached zero.

Based on the five temporal events, the gait initiation process was divided into four phases: P1 (the early stage of APA), P2 (the late stage of APA), P3 (the single support phase), and P4 (the double support phase), with detailed information presented in Figure 1B.

MATLAB 2020a (MathWorks, Natick, MA, United States) was used to compute the following parameters: ① the loading percentage on the paretic limb prior to gait initiation (*F*_p_); ② the durations of P1 to P4 (Δ*t*_P1_, Δ*t*_P2_, Δ*t*_P3_, Δ*t*_P4_) and the total gait initiation duration (Δ*t*_total_, from T0 to T4); ③ initial stance width, defined as the mediolateral distance between the heels during quiet standing prior to gait initiation; ④ step length and step width, defined, respectively, as the anteroposterior and mediolateral distances between the heels at leading limb contact; ⑤ COP displacement in the anteroposterior (DCOPX) and mediolateral (DCOPZ) directions during P1, P2, and P3, represented as DCOPX1, DCOPX2, DCOPX3 and DCOPZ1, DCOPZ2, DCOPZ3, respectively, with DCOPZ1 and DCOPZ2 normalized to the initial stance width; ⑥ the peak (*F*_xmax_) and impulse (Impulse_x_) of the anteroposterior ground reaction force (*F*_x_), calculated separately for each lower limb before toe-off of the respective limb. Impulse_x_ was computed as the area under the *F*_x_-time curve. *F*_xmax_-lead and Impulse_x_-lead denote the *F*_xmax_ and Impulse_x_ generated by the leading limb, while *F*_xmax_-trail and Impulse_x_-trail denote those generated by the trailing limb.

All ground reaction forces and impulses were normalized to body weight and expressed as percentages (%BW). For consistency, positive force and COP displacement were defined as anterior along the anteroposterior axis and toward the leading limb along the mediolateral axis; negative values were defined as posterior and toward the trailing limb, respectively.

To reduce the influence of left-right limb differences on group comparisons, healthy individuals were grouped based on the hemiplegic side of their matched stroke patients. If the patient had right-sided hemiplegia, the matched healthy individual’s right limb was treated as the matched paretic side and the left as the matched non-paretic side; this assignment was reversed for left-sided hemiplegia.

The average of three trials was used for statistical analyses. To evaluate within-session reliability, intraclass correlation coefficients (ICCs) were calculated across the three trials for each parameter. Overall, most variables demonstrated acceptable to good reliability, except for a few parameters. All statistical analyses were performed using R software (version 4.4.2; R Foundation for Statistical Computing, Vienna, Austria). Both between-group and within-group comparisons were conducted for the control and stroke groups. During between-group comparisons, gait initiation with the paretic and non-paretic limbs in the stroke group was compared with the matched paretic and matched non-paretic limb initiations in the control group, respectively. Correlation analyses were also performed between gait initiation parameters and clinical assessments within the stroke group.

For normally distributed parameters, data were presented as the mean and standard deviation (mean ± SD); independent and paired *t*-tests were used for between-group and within-group comparisons, respectively. Non-normally distributed parameters were presented as the median and interquartile range (IQR); the Mann–Whitney *U* test and Wilcoxon signed-rank test were used for between-group and within-group comparisons, respectively. Pearson and Spearman correlation analyses were applied to normally and non-normally distributed data, respectively. A *p*-value of <0.05 was considered statistically significant. *Post hoc* power analyses were performed using G*Power (version 3.1) to evaluate the adequacy of the sample size. With 34 participants per group and *α* = 0.05, the calculated power (1 – *β*) exceeded 0.80 for both between-group comparisons and correlational tests, indicating sufficient statistical power.

## 3 Results

### 3.1 Participant characteristics

Demographic and clinical characteristics of the participants are summarized in Table 1. No significant differences were observed between the stroke and control groups in age (48.26 ± 12.23 vs. 48.15 ± 12.57 years, *p* = 0.969), height (168.62 ± 7.28 vs. 168.56 ± 8.08 cm, *p* = 0.975), and weight (71.83 ± 11.71 vs. 70.63 ± 11.87 kg, *p* = 0.675). Among the stroke participants, 25 had ischemic strokes and 9 had hemorrhagic strokes. Hemiparesis was evenly distributed between the right and left sides (*n* = 17 per side). The median time since stroke onset was 2.33 months (IQR: 1.29–4.23). The mean FMA-LE score was 26.32 ± 4.82. The median TUG time was 19.29 s (IQR: 11.21–32.13), and the median 6MWT was 180.00 meters (IQR: 76.73–388.38).

### 3.2 Spatiotemporal parameters and center of pressure (COP) displacement

#### 3.2.1 Between-group comparisons

As summarized in Table 2, Δ*t*_P2_, Δ*t*_P4_, and Δ*t*_total_ were significantly prolonged in the stroke group under both paretic and non-paretic limb initiation conditions compared to controls (*p* < 0.001). Whereas Δ*t*_P3_ was increased during paretic limb initiation but reduced during non-paretic limb initiation compared to controls (*p* < 0.001). No significant between-group differences were observed for Δ*t*_P1_ and the initial stance width (*p* > 0.05). In addition, the stroke group exhibited shorter step length, and greater step width under both initiation conditions compared to controls (*p* < 0.01).

**Table 2:** Spatiotemporal parameters and COPX in the stroke and control groups.

Variable	Initiation with (or matched) paretic limb			Initiation with (or matched) non-paretic limb		
	Stroke	Control	*p*-value	Stroke	Control	*p*-value
Δ*t*_P1_ (s)	0.20 (0.17, 0.26)	0.18 (0.16, 0.21)	0.162	0.18(0.12, 0.24)	0.19 (0.16, 0.21)	0.589
Δ*t*_P2_ (s)	0.46 (0.37, 0.58)	0.33 (0.30, 0.38)	<0.001	0.58(0.43, 0.79)*	0.35 (0.30, 0.38)	<0.001
Δ*t*_P3_ (s)	0.55 ± 0.16	0.40 ± 0.04	<0.001	0.31 ± 0.11*	0.41 ± 0.04	<0.001
Δ*t*_P4_ (s)	0.43 (0.27, 0.70)	0.18 (0.16, 0.22)	<0.001	0.37 (0.28, 0.53)	0.18 (0.16, 0.20)	<0.001
Δ*t*_total_ (s)	1.65 (1.38, 2.10)	1.09 (1.06, 1.18)	<0.001	1.47 (1.33, 1.76)*	1.12 (1.04, 1.19)	<0.001
Step length (cm)	30.12 ± 11.53	56.31 ± 5.84	<0.001	28.79 ± 14.30	56.61 ± 6.61	<0.001
Initial stance width (cm)	19.70 ± 3.12	18.32 ± 3.45	0.087	19.30 ± 2.78	18.80 ± 3.38	0.508
Step width (cm)	17.37 ± 3.40	15.08 ± 3.39	0.007	18.53 ± 4.05	15.66 ± 3.50	0.003
DCOPX1 (cm)	−0.38 (−0.91, 0.07)	−2.68 (−3.09, −2.09)	<0.001	−0.18 (−1.08, 0.04)	−2.73 (−3.23, −2.12)	<0.001
DCOPX2 (cm)	0.69 ± 2.25	0.82 ± 2.72	0.831	−0.17 ± 2.04	0.83 ± 2.98	0.111
DCOPX3 (cm)	2.07 (0.67, 8.13)	10.45 (9.25, 11.49)	<0.001	2.03 (0.69, 6.18)	11.25 (9.62, 12.40)	<0.001
DCOPZ1	0.12 (0.05, 0.18)	0.18 (0.13, 0.22)	<0.001	0.09 (0.04, 0.18)	0.15 (0.13, 0.19)	0.005
DCOPZ2	−0.70 ± 0.12	−0.80 ± 0.11	<0.001	−0.84 ± 0.13*	−0.77 ± 0.06	0.003
DCOPZ3 (cm)	−0.68 (−1.85, −0.17)	−2.48 (−3.41, −1.67)	<0.001	−0.67 (−2.18, 0.28)	−2.52 (−3.15, −1.90)	<0.001

*Indicates a statistically significant difference within the group (*p* < 0.05). No significant within-group differences were observed in the control group. Data are presented as mean ± standard deviation (SD) for normally distributed variables, and as median (interquartile range, IQR) for non-normally distributed variables. Δ*t*_P1_, Δ*t*_P2_, Δ*t*_P3_, Δ*t*_P4_ and Δ*t*_total_ represented the durations of P1 to P4 and the total gait initiation duration, respectively. DCOPX1, DCOPX2 and DCOPX3, as well as DCOPZ1, DCOPZ2, and DCOPZ3, represented the anteroposterior (COPX) and mediolateral (COPZ) center of pressure displacement during P1, P2, and P3, respectively, with DCOPZ1 and DCOPZ2 normalized to the initial stance width.

For COP displacement, posterior DCOPX1, forward DCOPX3, DCOPZ1 (toward the leading limb) and DCOPZ3 (toward the trailing limb) were all significantly reduced in the stroke group under both initiation conditions compared to controls (*p* < 0.01). However, DCOPZ2 (toward the trailing limb) was reduced during paretic limb initiation but increased during non-paretic limb initiation compared to controls (*p* < 0.01). DCOPX2 showed no significant difference between two groups.

#### 3.2.2 Within-group comparisons

Within-group comparisons in the stroke group revealed significant differences exclusively in Δ*t*_P2_, Δ*t*_P3_, Δ*t*_total_, and DCOPZ2. Specifically, compared to non-paretic limb initiation, paretic limb initiation exhibited shorter Δ*t*_P2_, longer Δ*t*_P3_ and Δ*t*_total_ in spatiotemporal parameters, and smaller DCOPZ2 in COP displacement (*p* < 0.05).

No significant differences were found between initiation conditions in the control group (*p* > 0.05).

### 3.3 Peak (*F*_xmax_) and impulse (Impulse_x_) of anteroposterior ground reaction force

As shown in Table 3, stroke patients demonstrated significantly reduced initial loading percentage on the paretic limb (*F*_p_) prior to gait initiation compared to controls (*p* < 0.001), with an average of approximately 44%. Moreover, the stroke group exhibited significantly reduced *F*_xmax_-lead, *F*_xmax_-trail, Impulse_x_-lead, and Impulse_x_-trail in both the paretic and non-paretic limbs compared to controls (*p* < 0.001).

**Table 3:** Peak force (*F*_xmax_) and impulse (Impulse_x_) of the anteroposterior ground reaction force in the leading and trailing limbs of stroke and control groups.

Variable	Initiation with (or matched) paretic limb			Initiation with (or matched) non-paretic limb		
	Stroke	Control	*p* value	Stroke	Control	*p* value
*F*_p_ (%)	44.55 ± 6.39	49.35 ± 2.65	<0.001	44.45 ± 6.62	49.54 ± 2.61	<0.001
*F*_xmax_-lead (%BW)	0.65 ± 0.80	4.85 ± 1.79	<0.001	2.11 ± 0.98*	4.60 ± 1.69	<0.001
Impulse_x_-lead (%BW·s)	−0.12 ± 0.45	1.43 ± 0.46	<0.001	0.66 ± 0.58*	1.43 ± 0.50	<0.001
*F*_xmax_-trail (%BW)	6.30(5.37, 9.45)	17.92 (14.95, 20.87)	<0.001	7.05 (4.02, 8.88)	18.87 (15.78, 20.42)	<0.001
Impulse_x_-trail (%BW·s)	4.75 ± 1.63	8.49 ± 0.96	<0.001	2.97 ± 2.66*	8.55 ± 0.91	<0.001

*Indicates a statistically significant difference within the group (*p* < 0.05). Data are presented as mean ± standard deviation (SD) for normally distributed variables, and as median (interquartile range, IQR) for non-normally distributed variables. *F*_p_: Initial paretic or matched paretic limb loading percentage (%body weight). *F*_xmax_-lead, Impulse_x_-lead, *F*_xmax_-trail, and Impulse_x_-trail represented the peak anteroposterior force and impulse values of the leading and trailing limbs, respectively. *F*_xmax_ and Impulse_x_ values are expressed as %BW and %BW·s, respectively (normalized to body weight in Newtons).

In the stroke group, the *F*_xmax_-lead, Impulse_x_-lead, and Impulse_x_-trail of the paretic limb were significantly smaller than those of the non-paretic limb (*p* < 0.05). However, no significant difference in *F*_xmax_-trail was observed between the paretic and non-paretic limbs (*p* > 0.05). No significant differences were found between two limbs in the control group (*p* > 0.05).

### 3.4 Correlation between COP displacement and clinical assessments

**Figure 2:**
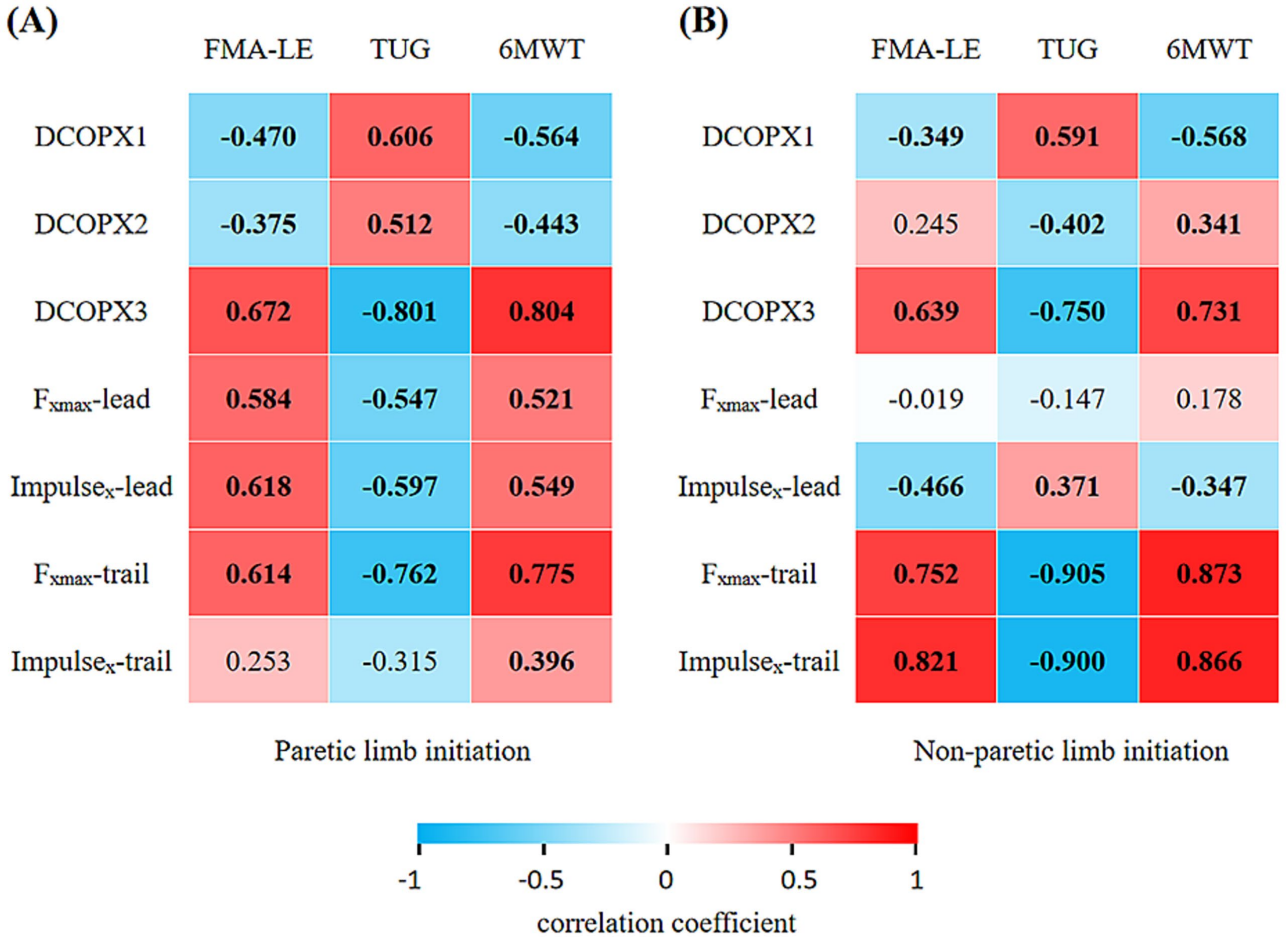
Correlation heatmaps between gait initiation parameters and clinical assessments under paretic and non-paretic limb initiation conditions: **(A)** Paretic limb initiation and **(B)** non-paretic limb initiation. Cells display correlation coefficients (*r*), with color indicating direction and magnitude (blue = negative, red = positive; scale −1 to 1). DCOPX1, DCOPX2, and DCOPX3 denote anteroposterior center of pressure displacement (COPX) during phases P1, P2, and P3, respectively. *F*_xmax_-lead and Impulse_x_-lead denote *F*_xmax_ and Impulse_x_ of the leading limb, while *F*_xmax_-trail and Impulse_x_-trail denote those of the trailing limb. FMA-LE, Fugl-Meyer Assessment for the Lower Extremity; TUG, Timed Up and Go Test; 6MWT, 6-Minute Walk Test. Values in bold indicate statistically significant correlations (*p* < 0.05).

**Figure 3:**
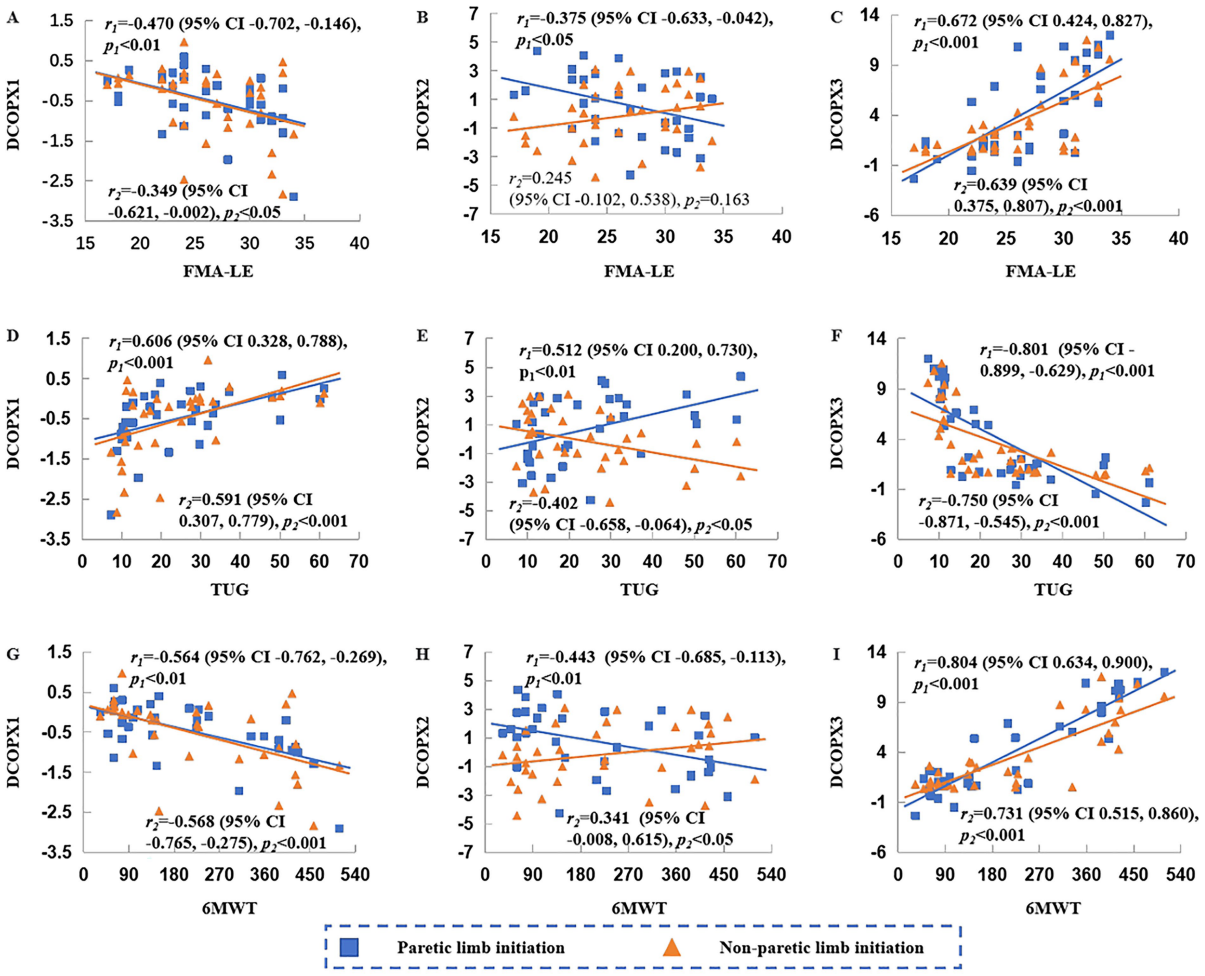
Correlations between anteroposterior center of pressure displacement (COPX) and clinical assessments. DCOPX1, DCOPX2, and DCOPX3 denote COPX during phases P1, P2, and P3, respectively. FMA-LE, Fugl-Meyer Assessment for the lower extremity; TUG, Timed Up and Go Test; 6MWT, 6-Minute Walk Test. Plots **A, B**, and **C** show the correlations between DCOPX1, DCOPX2, and DCOPX3 with FMA-LE, respectively; Plots **D, E**, and **F** show the correlations between DCOPX1, DCOPX2, and DCOPX3 with TUG, respectively; Plots **G,H**, and **I** show the correlations between DCOPX1, DCOPX2, and DCOPX3 with 6MWT, respectively. *r*1 and *r*2 denote correlation coefficients under paretic and non-paretic limb initiation condition, respectively; *p*1 and *p*2 indicate the corresponding significance levels.

As shown in Figures 2, 3, DCOPX1 was negatively correlated with FMA-LE scores (*r* = −0.349 to −0.470, *p* < 0.05) and 6MWT (*r* = −0.564 to −0.568, *p* < 0.01), and positively correlated with TUG time (*r* = 0.591 to 0.606, *p* < 0.001) under both initiation conditions. DCOPX3 showed significant positive correlations with FMA-LE scores (*r* = 0.639 to 0.672, *p* < 0.001) and 6MWT (*r* = 0.731 to 0.804, *p* < 0.001), and negative correlations with TUG time (*r* = −0.750 to −0.801, *p* < 0.001) under both initiation conditions.

When gait was initiated with the paretic limb, DCOPX2 was negatively correlated with FMA-LE scores (*r* = −0.375, *p* < 0.05) and 6MWT (*r* = −0.443, *p* < 0.01), and positively correlated with TUG time (*r* = 0.512, *p* < 0.01). Under the non-paretic limb initiation condition, DCOPX2 was negatively correlated with TUG time (*r* = −0.402, *p* < 0.05), positively correlated with 6MWT (*r* = 0.341, *p* < 0.05), and showed no significant correlation with FMA-LE.

### 3.5 Correlations between *F*_xmax_, Impulse_x_, and clinical assessments

**Figure 4:**
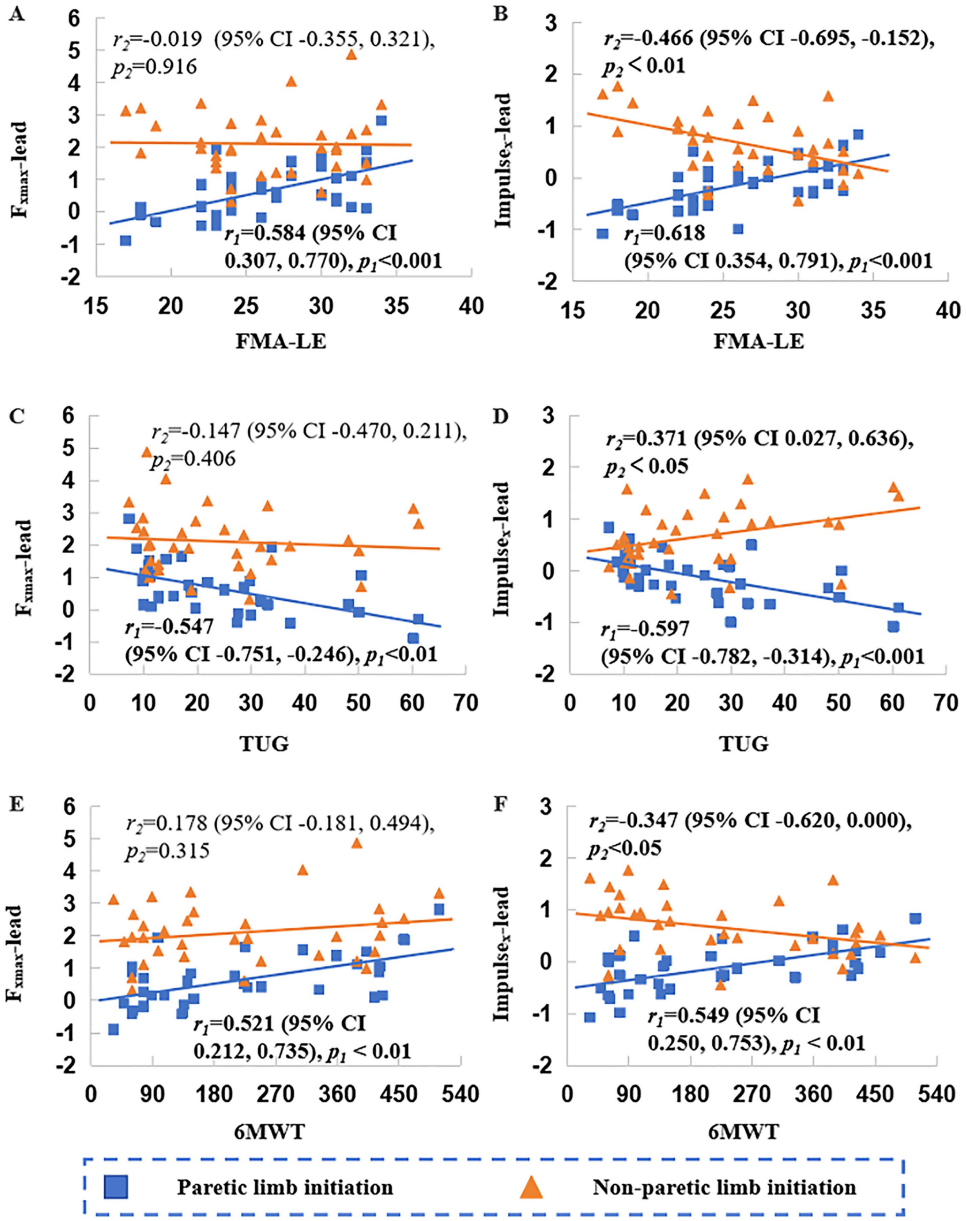
Correlations of peak force (*F*_xmax_) and impulse (Impulse_x_) of the anteroposterior ground reaction force (*F*_x_) in the leading limb with clinical assessments. FMA-LE, Fugl-Meyer Assessment for the Lower Extremity; TUG, Timed Up and Go Test; 6MWT, 6-Minute Walk Test. Plots **A, C**, and **E** show correlations between Fxmax with FMA-LE, TUG, and 6MWT, respectively; plots **B, D**, and **F** show correlations between *Impulse_x_* with FMA-LE, TUG, and 6MWT, respectively. *r*_1_ and *r*_2_ denote correlation coefficients under paretic and non-paretic limb initiation condition, respectively; *p*_1_ and *p*_2_ indicate the corresponding significance levels.

**Figure 5:**
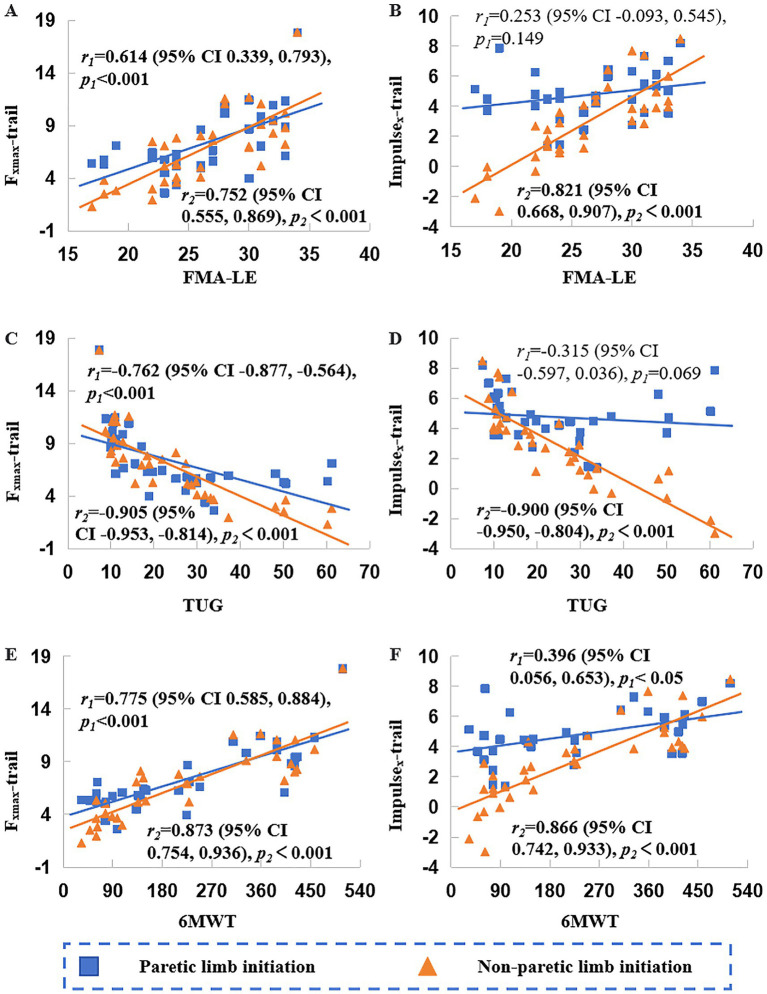
Correlations of peak force (*F*_xmax_) and impulse (Impulse_x_) of the anteroposterior ground reaction force (*F*_x_) in the trailing limb with clinical assessments. FMA-LE, Fugl-Meyer Assessment for the Lower Extremity; TUG, Timed Up and Go Test; 6MWT, 6-Minute Walk Test. Plots **A, C**, and **E** show correlations between Fxmax and FMA-LE, TUG, and 6MWT, respectively; plots **B, D**, and **F** show correlations between *Impulse_x_* and FMA-LE, TUG, and 6MWT, respectively. *r*_1_ and *r*_2_ denote correlation coefficients under paretic and non-paretic limb initiation condition, respectively; *p*_1_ and *p*_2_ indicate the corresponding significance levels.

As shown in Figures 2, 4, 5, for the paretic limb, *F*_xmax_-lead, Impulse_x_-lead, *F*_xmax_-trail, and Impulse_x_-trail were positively correlated with the FMA-LE (*r* = 0.584 to 0.821, *p* < 0.001) and 6MWT (*r* = 0.521 to 0.873, *p* < 0.01), and negatively correlated with the TUG time (*r* = −0.547 to −0.905, *p* < 0.01).

For the non-paretic limb, *F*_xmax_-lead showed no significant correlations with all assessments. Impulse_x_-lead was negatively correlated with FMA-LE scores (*r* = −0.466, *p* < 0.01) and 6MWT (*r* = −0.347, *p* < 0.05), and positively correlated with TUG time (*r* = 0.371, *p* < 0.05). *F*_xmax_-trail was positively correlated with FMA-LE (*r* = 0.614, *p* < 0.001) and 6MWT (*r* = 0.775, *p* < 0.001), and negatively correlated with TUG time (*r* = −0.762, *p* < 0.001). Impulse_x_-trail was only positively correlated with 6MWT (*r* = 0.396, *p* < 0.05).

## 4 Discussion

This study used a refined phase segmentation to characterize gait initiation across the full initiation cycle under both paretic and non-paretic limb initiation conditions and to identify stage-specific abnormalities that may be overlooked in prior studies. Using this framework, we found that APA prolongation in stroke was mainly driven by the late APA subphase (P2), whereas reduced posterior COP displacement during APA was primarily attributable to the early subphase (P1). Moreover, the longer total initiation time during paretic-limb initiation was chiefly explained by a prolonged single-support phase (Δ*t*_P3_). Importantly, beyond the APA phase, we examined COP displacement across the full initiation cycle: consistent with previous reports, posterior DCOPX1 was reduced, and we further observed reduced DCOPX3 and DCOPZ3 during single support under both initiation conditions. These findings provide additional evidence for a more comprehensive understanding of gait-initiation control strategies in stroke patients. In addition, multiple COP- and kinetic-related measures were associated with clinical scores, with particularly strong associations for DCOPX1/DCOPX3 and paretic-limb propulsion-related metrics, supporting their clinical relevance and utility for guiding rehabilitation targets.

Stroke patients frequently exhibit prolonged total initiation time and reduced step length during gait initiation, as reported in previous studies (15–17). Consistent with these findings, the present study identified similar impairments, and additionally observed increased step width during gait initiation—a pattern also noted during steady-state walking (24). To our knowledge, increased step width during gait initiation has been rarely reported in prior studies. This widened step width may represent a compensatory mechanism to enhance stability in response to postural instability. Moreover, our findings also revealed a significant prolongation in total initiation time during paretic limb initiation compared to non-paretic limb initiation. With the refined phase segmentation, we further identified prolonged single-limb support time (Δ*t*_P3_) during paretic limb initiation as the primary contributor to this temporal asymmetry, a finding that has not been explicitly reported in previous studies. This finding may reflect impaired weight-bearing capacity on the paretic side and compensatory reliance on the non-paretic limb. During non-paretic limb initiation, the single-limb support phase on the paretic side represented the most unstable period and was often shortened to reduce fall risk. During paretic limb initiation, the Δ*t*_P3_ on the non-paretic limb was prolonged, which may result from two factors: difficulty in swinging the paretic limb, and increased reliance on the non-paretic limb for support and propulsion.

Furthermore, the study demonstrated that, stroke patients exhibited prolonged Δ*t*_P2_ and Δ*t*_P4_ compared to controls, indicating impaired inter-limb weight transfer. Within-group comparisons revealed a longer Δ*t*_P2_ during non-paretic limb initiation, suggesting greater difficulty in transferring weight onto the paretic limb. The longer Δ*t*_P2_ during non-paretic limb initiation may also be attributed to asymmetrical weight-bearing. Patients often bear more weight on the non-paretic limb prior to initiation (25), a phenomenon also observed in our study, leading to a prolonged weight transfer toward the paretic limb.

During the APA phase, the COP initially shifts laterally and posteriorly toward the leading limb, followed by a shift toward the trailing limb (4). Previous studies often treated the APA phase as a single unit, and reported prolonged duration and reduced posterior COP displacement during the APA phase in stroke patients (14, 15). However, because APA comprises two directionally distinct weight-shift processes, such aggregation may obscure sub-phase-specific abnormalities. To facilitate phase-specific analysis, the APA phase was further divided into P1 and P2 using the refined phase segmentation proposed in this study, corresponding to COP movement toward the leading and trailing limbs, respectively. Our study further revealed that the previously reported prolongation of the APA phase was primarily attributable to the extension of its late stage (P2), as Δ*t*_P1_ showed no significant differences, while Δt_P2_ differed both between and within groups. However, the reduction in COP displacement during the APA phase was primarily due to decreased displacement in its first stage (P1), since DCOPX1 was reduced, whereas no differences were observed in DCOPX2. Such stage-specific information would not be obtainable if the APA phase were analyzed as a single unit, as is commonly done in the current literature. From a clinical perspective, prolonged P2 may reflect reduced efficiency of mediolateral weight transfer during the late APA period; therefore, rehabilitation may emphasize inter-limb mediolateral weight-shift training during this time window. Reduced COPX1 displacement during P1 may suggest that greater attention should be given to facilitating tibialis anterior activation and mitigating triceps surae spasticity during this phase, as previous studies have also suggested that reduced COP displacement during the APA phase after stroke may be related to decreased tibialis anterior activation and insufficient inhibition of the triceps surae.

Most studies have focused on the APA phase, and relatively few have examined the entire gait-initiation cycle. Evidence remains limited regarding COP displacement changes across the entire initiation cycle under both paretic and non-paretic limb initiation conditions. In this study, using our refined full-cycle phase segmentation, we systematically characterized stage-specific COP displacement patterns across the entire gait-initiation cycle under both initiation conditions, thereby providing evidence for a more comprehensive understanding of gait-initiation control strategies in stroke patients.

The reduction of COPX1 and COPZ1 displacements observed in the present study among stroke patients may be related to abnormal lower limb muscle activation patterns, such as insufficient activation of the tibialis anterior, inadequate inhibition of the soleus and gastrocnemius muscles, and insufficient activation of the hip abductor and adductor muscles (26–29). Our results showed that stroke patients exhibited reduced DCOPZ2 during paretic limb initiation, and increased DCOPZ2 during non-paretic limb initiation, compared to controls, which is consistent with earlier studies reporting a similar mediolateral COP displacement pattern during the APA phase after stroke (15, 17). This asymmetry may be attributed to asymmetric weight-bearing prior to gait initiation, which shifted the COP closer to the non-paretic limb. Therefore, greater displacement was required for COP to reach the paretic limb when non-paretic limb initiation. Conversely, the opposite pattern was observed during paretic limb initiation.

Compared to controls, stroke patients exhibited reduced forward DCOPX3 and lateral DCOPZ3, regardless of the initiating limb, consistent with findings reported in steady-state walking (30, 31). When the paretic limb served as the supporting limb, the reduced COP displacement may suggest challenges in controlling forward progression during single-support over the paretic limb, and may also be related to altered plantar pressure distribution. Specifically, stroke patients often exhibit increased rear-foot loading and reduced forefoot pressure (32, 33), resulting in a more posterior COP position and reduced forward displacement at the end of single support, which is consistent with the limited forefoot COP progression during steady-state walking reported previously (30, 31). When the non-paretic limb was the supporting limb, reduced COP displacement may result from the difficulty in swing paretic limb or a compensatory strategy to avoid excessive postural shifts that could increase fall risk.

Consistent with previous studies, the present study demonstrated that stroke patients exhibited reduced peak anteroposterior ground reaction force (*F*_xmax_) and propulsive impulse (Impulse_x_) compared to controls, with more pronounced impairments observed on the paretic side (16, 18). These findings suggest that the forward propulsion capacity of the paretic limb may be an important target for rehabilitation. The diminished propulsion observed in the paretic limb may be attributed to reduced muscle strength in ankle plantar flexors and hip extensors (18, 34). With respect to the non-paretic side, reduced propulsion may be explained by three factors. First, although stroke primarily affects the contralesional limb, the ipsilesional limb may also exhibit subtle neuromuscular impairment due to damage to ipsilesional descending motor pathways (e.g., uncrossed corticospinal, reticulospinal, vestibulospinal tracts) (34, 35). Second, prolonged inactivity may lead to disuse atrophy and muscle weakness. Third, stroke patients may intentionally limit non-paretic push-off to prioritize postural stability and reduce fall risk (18).

Evidence linking gait-initiation biomechanics to clinical scales remains sparse. To the best of our knowledge, only one study has examined whether maximal posterior COP displacement during the APA phase and paretic-limb latency to peak COP displacement are associated with clinical outcomes (19). Whether COP displacement in later phases, as well as key kinetic variables, relates to commonly used scales has remained unclear. To address this gap, we examined associations between multiple stage-specific COP displacement measures and kinetic parameters across the full initiation cycle and widely used clinical assessments.

Correlation analysis revealed that greater posterior DCOPX1 and anterior DCOPX3 were associated with higher FMA-LE scores and longer 6MWT distance, and shorter TUG time. These findings suggest that larger COP shifts are associated with better functional performance, and that DCOPX1 and DCOPX3 may serve as potential indicators of clinical motor performance. Importantly, such precise biomechanical parameters can compensate for the limitations of traditional clinical scales in capturing underlying biomechanical mechanisms and providing objective, quantitative assessment. It is worth noting that, given the cross-sectional design, these findings should not be taken as evidence of causality between DCOPX1/DCOPX3 and clinical motor function.

However, COPX2 was only weakly correlated with TUG time and 6MWT under both initiation conditions, and the direction of correlation varied depending on the initiating limb. As illustrated in Figure 3, patients with poorer functional performance tended to exhibit greater anterior DCOPX2 during paretic limb initiation, but posterior displacement when initiating with the non-paretic limb. This pattern may be related to a more posterior plantar pressure distribution under the paretic foot in more severely impaired individuals, resulting in a more posterior COP position (32, 33). During paretic limb initiation, weight transfer from this relatively posterior position to the non-paretic limb during P2 may contribute to increased anterior DCOPX2. Conversely, during non-paretic-limb initiation, COP transfer toward the paretic stance limb may yield a relatively more posterior DCOPX2 displacement. This condition-dependent difference appears more evident in lower-functioning participants (as suggested by the scatterplots; e.g., FMA-LE ≤ 20, TUG ≥ 40 s, and 6MWT ≤ 110 m) and less apparent in higher-functioning participants, which may partly explain why DCOPX2 did not differ significantly between initiation conditions when analyzing the entire cohort. Overall, DCOPX2 may reflect anterior-posterior adjustments during weight transfer from the leading to the trailing limb and may provide indirect information regarding impairment-related plantar pressure distribution. Accordingly, it may also serve as a partial indicator of impairment severity; when interpreted alongside other gait-initiation parameters, it may provide a more comprehensive view.

As illustrated in Figures 4, 5, all kinetic parameters of the paretic limb demonstrated significant and consistent correlations with clinical assessments, particularly when the paretic limb served as the supporting leg. In contrast, only a subset of the non-paretic limb parameters exhibited weaker and less consistent associations. These findings underscore the potential value of propulsion-related metrics from the paretic limb as objective markers linked to clinical performance and highlight paretic limb propulsion as a critical target in rehabilitation. In general, greater force and impulse values were associated with better functional performance. However, an exception was observed for the Impulse_x_-lead of the non-paretic limb, where higher values were associated with poorer clinical outcomes. However, given the relatively low ICC of this parameter, this result should be interpreted with caution.

Several limitations should be noted. First, the small sample size may limit further subgroup analyses. Second, although most gait initiation parameters demonstrated acceptable to good within-session reliability, a few variables showed relatively low ICC values (Δ*t*_P1_, non-paretic Impulse_x_-lead and Impulse_x_-trail in the stroke group). Third, participants were not strictly matched based on their preferred leading limb in this study. In addition, we did not examine whether absolute GRF magnitude influenced the kinematic outcomes. Future research should include larger samples, increase the number of repeated trials, and consider swing-limb preference as well as the influence of absolute GRF magnitude on kinematic outcomes in the study design and analyses.

## 5 Conclusion

Based on the refined phase-segmentation approach, we obtained additional insights: during gait initiation, stroke patients exhibited impaired APA capacity, which was primarily reflected by a prolonged P2 duration and reduced COP displacement in P1. COPX1, COPX3, and paretic-limb propulsive force may serve as potential objective, quantitative indicators for clinical assessment. Gait initiation training should be considered for incorporation into post-stroke rehabilitation, with particular attention to improving weight shifting, paretic-limb weight-bearing, and propulsion.

## Data Availability

The original contributions presented in the study are included in the article/Supplementary material, further inquiries can be directed to the corresponding author.
