# Supplementary material for: Biomechanical analysis of gait initiation in stroke survivors based on a modified phase segmentation method
Source: Front Neurol. 2026 Feb 9;17:1716860. doi: 10.3389/fneur.2026.1716860 (PMC12926147; doi:10.3389/fneur.2026.1716860)
Supplement: Supplementary file 1 [file Table_1.DOCX]

The supplementary figure helps readers better understand the characteristics of COP trajectories in healthy controls and stroke survivors under paretic- and non-paretic-limb initiation conditions. Panel (A) shows a healthy subject initiating gait with the right limb; panel (B) shows a right hemiparetic patient initiating gait with the paretic limb; and panel (C) shows the same patient initiating gait with the non-paretic limb. The x-axis represents COPZ (m) (mediolateral COP position), and the y-axis represents COPX (m) (anteroposterior COP position). T0, T1, T2, and T3 denote the temporal events of initiation onset, the most lateral COP shift toward the swing limb, leading-limb toe-off, and leading-limb strike, respectively.

The figure title and caption are as follows:

Figure S1 Center of pressure(COP) trajectory in the anteroposterior(COPX) and mediolateral(COPZ) direction of a healthy subject initiating with the right limb (A) ; a right hemiparetic patient initiating with the paretic limb(B) and non-paretic limb (C); T0, T1, T2, and T3 indicate the temporal events of onset of initiation, COP most lateral toward the swing limb, leading limb toe-off, and leading limb strike, respectively.
