# Supplementary figures and images for: Biomechanical analysis of gait initiation in stroke survivors based on a modified phase segmentation method
Source: Front Neurol. 2026 Feb 9;17:1716860. doi: 10.3389/fneur.2026.1716860 (PMC12926147; doi:10.3389/fneur.2026.1716860)

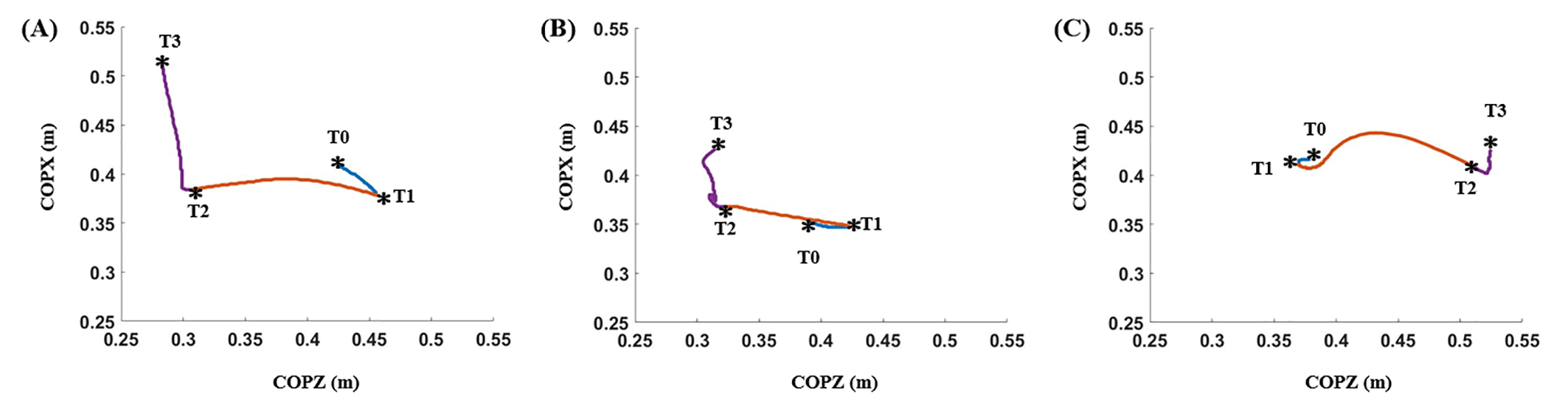

Supplement: Supplementary file 3 [file Image_1.TIF]
